# Staff Changes at Camberwell Infirmary

**Published:** 1913-12-20

**Authors:** 


### STAFF CHANGES AT CAMBERWELL INFIRMARY.

In addition to Dr. G. C. Soutter, assistant medical officer at Camberwell Infirmary, who, as reported in The Hospital, has resigned, Dr. A. Mearns, junior medical officer, has sent in his resignation. The Guardians have decided to promote Dr. C. H. L. Rixon, third assistant medical officer, to the post occupied by Dr. Soutter, and to appoint Dr. James Hill, who was previously house surgeon and ophthalmic officer at the Royal Infirmary, Sheffield, second assistant medical officer. The board has decided to advertise the vacant position of third assistant medical officer.

